# 1‐Aryl‐6,7‐Dimethoxy‐3,4‐Dihydroisoquinoline‐2(1*H*)‐Sulfonamides as hCA XII Selective Inhibitors: Experimental and Theoretical Studies to Interrogate the Isoform Selectivity

**DOI:** 10.1002/cmdc.70390

**Published:** 2026-07-23

**Authors:** Federico Ricci, Anna Di Fiore, Andrea Angeli, Laura De Luca, Francesca Mancuso, Davide Esposito, Giuseppina De Simone, Claudiu T. Supuran, Rosaria Gitto

**Affiliations:** ^1^ CHIBIOFARAM Department University of Messina Messina Italy; ^2^ Institute of Biostructures and Bioimaging CNR Napoli Italy; ^3^ NEUROFARBA Department University of Florence Sesto Fiorentino Florence Italy

**Keywords:** cancer, carbonic anhydrase, chemistry, crystallography, docking (molecular), drug design, extracellular, medicinal chemistry, rational design

## Abstract

Human carbonic anhydrase XII (hCA XII) represents an important pharmacological target for different types of cancer. hCA XII plays a crucial role in regulating both extracellular and intracellular pH, thereby influencing cancer cell proliferation, invasion, growth, and metastasis. Although the interaction features of hCA inhibitors (hCAIs) with the catalytic site of distinct hCA isoforms are generally well described, the lack of selectivity remains a major challenge. In a previous work, we have reported a series of 1‐aryl‐6,7‐dimethoxy‐3,4‐dihydroisoquinoline‐2(1*H*)‐sulfonamides displaying weak activity against the ubiquitous hCA I and hCA II, thus emerging as hCAIs that may be free of unwanted side‐effects. Herein, further evaluation of their CA inhibitory effects allowed to disclose three potent hCA XII inhibitors at low nanomolar concentrations (*K*_i_ values ranging from 5.5 to 6.5 nM). Additionally, they showed remarkable isoform selectivity compared with the well‐known inhibitor SLC‐0111, currently in clinical trials as an antitumor agent. Crystallography analyses and computational studies clarified the molecular basis of this behavior and provided valuable insights for the rational design of selective inhibitors targeting hCA XII.

## Graphical abstract

Experimental and theoretical studies have disclosed the more relevant structural requirements for effective inhibition and an impressing selectivity toward tumor‐expressed hCA XII isoform over hCA II.

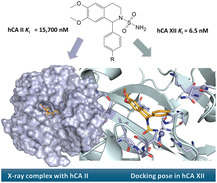

## 1 Introduction

Carbonic anhydrases (CAs) are ubiquitous metalloenzymes present in various organisms including animals, bacteria, and fungi. They catalyze the reversible carbon dioxide hydration reaction with formation of bicarbonate and a proton, ensuring a fine pH regulation and controlling various physiological processes. Human (h) CAs belong to the α‐class and comprise fifteen different isoforms (hCA I–XIV including the two mitochondrial hCA VA and hCA VB isoforms) each with distinct tissue and cellular distribution [1]. It has been well‐established that the various hCAs can be involved in several human pathologies. For this reason, in recent years there has been a growing interest in hCAs as targets for the design of inhibitors (hCAIs) with biomedical applications. However, the identification of isoform‐selective inhibitors is a particularly challenging task, mainly due to the high degree of sequence homology shared among the active sites of the 15 hCA isoforms.

**FIGURE 1:**
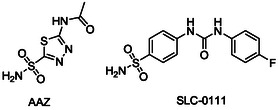
Chemical structures of hCA IX/hCA XII inhibitors acetazolamide (AAZ) and 4‐(3‐(4‐fluorophenyl)ureidobenzenesulfonamide (SLC‐0111, U‐104).

The most extensively studied hCAIs are small molecules (e.g., the prototype acetazolamide (AAZ) displayed in Figure 1), bearing a sulfonamide functional group capable of coordinating the zinc ion at the bottom of the hCA active site [2, 3]. These molecules also possess additional pharmacophoric elements that enable interactions with hydrophobic/hydrophilic residues lining the wall of the peculiar active site cavity characterizing each isoform [4, 5].

The search for new anti‐tumor drugs has been recently focused on the development of selective inhibitors of hCA IX and hCA XII; specifically these two isoforms that are overexpressed in several cancer cell types and play a key role in the regulation of the extracellular pH within the tumor microenvironment [6, 7, 8]. From a structural point of view, hCA IX and hCA XII are transmembrane proteins with their catalytic site exposed to the extracellular space. Several sulfonamide‐based hCA IX and hCA XII inhibitors have been developed so far; among them, the benzenesulfonamide derivative SLC‐0111 [9] (also named U‐104, Figure 1) has shown high selectivity and potency and has advanced to Phase IIb clinical trials for the treatment of metastatic solid tumors (https://clinicaltrials.gov/study/NCT03450018). Subsequently, many other benzenesulfonamides possessing high hCA IX/hCA XII affinity combined with relevant selectivity over hCA I/II were identified and characterized for their binding profile [10, 11, 12].

In recent years, our efforts to discover hCA inhibitors led to the identification of a series of 1‐aryl‐3,4‐dihydroisoquinoline‐2(1*H*)‐sulfonamides [13, 14, 15, 16]. Among them, several compounds displayed weak inhibition of hCA I, hCA II and hCA IX (*K*_i_ values in micromolar range) [14], but acted as efficient inhibitors of hCA VII (*K*_i_ values in nanomolar range) [15]; this latter evidence prompted us to expand our investigations and to validate the hypothesis that this chemotype could provide a certain degree of isoform selectivity toward druggable hCAs. Based on this assumption, we further advanced the characterization of selected compounds by testing their inhibitory properties against hCA XII as key mediator of cancer proliferation [17, 18, 19]. To elucidate the structural determinants of isoform selectivity, we carried out structural studies by analyzing the crystallographic structures of 1‐aryl‐6,7‐dimethoxy‐3,4‐dihydroisoquinoline‐2(1*H*)‐sulfonamides in complex with hCA II; subsequently, molecular docking studies were performed to compare their binding within the hCA II active site cavity with their hypothetical binding pose within hCA XII binding site.

## 2 Results and Discussion

### 2.1 Assesment of CA Inhibitory Effects toward hCA XII Isoform

**FIGURE 2:**
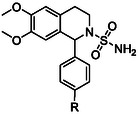
Chemical structures of the investigated 1‐aryl‐6,7‐dimethoxy‐3,4‐dihydroisoquinoline‐2(1*H*)‐sulfonamides (**1**, R = H), **2** (R = F) and **3** (R = NH_2_).

To begin our investigation on hCA XII, we focused on three 1‐aryl‐6,7‐dimethoxy‐3,4‐dihydroisoquinoline‐2(1*H*)‐sulfonamides (**1**–**3**, Figure 2). These compounds were previously reported to exhibit low inhibitory activity against hCA I, hCA II, hCA IX, and hCA XIV (*K*_i_ values > 3,000.00 nM) [13, 14, 20]; interestingly, they proved to efficiently inhibit hCA VII at low nanomolar concentration (*K*_i_ values ranging from 6.0 to 7.1 nM) [15]. A complete overview of previously reported data is provide in Table S1. Herein, we studied unsubstituted compound **1** (R = H) as well as related compounds **2** (R = F) and **3** (R = NH_2_), bearing distinct chemical functionalities at para‐position of the aryl substituent at C‐1 of isoquinoline core system; this very preliminary investigation was aimed at exploring the role of the nature of R substituent that could provided opposite electronic and polar effects. The selected compounds possess two hydrophobic aromatic moieties that may induce steric hindrance within the active‐site cavity of several hCA isoforms as well as the canonical zinc anchoring group, a key feature for all sulfonamide/sulfamide/sulfamate based hCAIs reported in literature [2, 3].

To evaluate the CA inhibitory effects against the target isoform hCA XII, compounds **1–3** were retrieved from our chemical repository named CHIME2024, where they were already available in‐stock of about 30 mg amount thus avoiding the need for resynthesis. Before carrying out the biochemical evaluation, the compounds **1–3** were explored to analyze their purity and stability. The chemical properties and spectral data of available racemic mixtures of **1–3** resulted in agreement with the proposed chemical structures [13, 14] as detailed in Experimental Section and Supporting Information.

The inhibitory activities against hCA XII were assessed using the stopped flow CO_2_ hydration assay [10, 21]. The obtained data are reported in Table 1, together with previously determined *K*_i_ values against the ubiquitous hCA II for comparative purpose [14]. The Table 1 also displayed the *K*_i_ values of the well‐known CA inhibitor AAZ, previously tested in the same experimental conditions [10, 21].

**TABLE 1:** *K*_i_ values (nM) against hCA II and hCA XII for tested compounds **1–3**, SLC‐0111 and reference compound AAZ.

		*K*_i_, nM^a^	
Code name	R	hCA II	hCA XII
**1**	H	15 700^b^	6.5
**2**	F	14 980^b^	6.5
**3**	NH_2_	11 300^b^	5.5
AAZ	—	12.1^b^	5.7^b^
SLC‐0111	—	960.0^c^	4.5^c^

a Errors in the range of ±5%–10% of the reported values, from 3 different assays.

b Data taken from Refs. [14, 20].

c Data taken from Ref. [9].

Interestingly, results revealed that compounds **1–3** were potent and selective inhibitors of hCA XII, with *K*_i_ values ranging from 5.5 to 6.5 nM. Overall, these inhibitors revealed a significant improvement of hCA XII selectivity over hCA II when compared to reference compound AAZ. Notably, all investigated compounds **1–3** demonstrated greater selectivity when compared to SLC‐0111 (*K*_i_ value of 960 nM against hCA II and *K*_i_ value of 4.5 nM against hCA XII). However, a flat structure–activity relationship (SAR) was observed; indeed the replacement of the phenyl ring of compound **1** with a 4′‐fluorophenyl (**2**) or 4′‐aminophenyl (**3**) moiety did not have any influence on the *K*_i_ value. On this basis, we decided not to further expand the SAR investigations by screening additional analogs from our CHIME2024 repository, bearing different substituents at the para‐position of the phenyl ring.

Given the interesting selectivity profile of these compounds, in the second step of this study, we in‐depth investigated their structural features, with the aim of elucidating their binding mode to ubiquitous hCA II in comparison with tumor‐expressed hCA XII. Consequently, we carried out a comprehensive experimental and computational study toward hCA II and hCA XII for compounds **1** and **2** that were studied as prototypes of 1‐aryl‐6,7‐dimethoxy‐3,4‐dihydroisoquinoline‐2(1*H*)‐sulfonamides.

### 2.2 X‐Ray Analysis of hCA II in Complex with compounds 1 and 2

**FIGURE 3:**
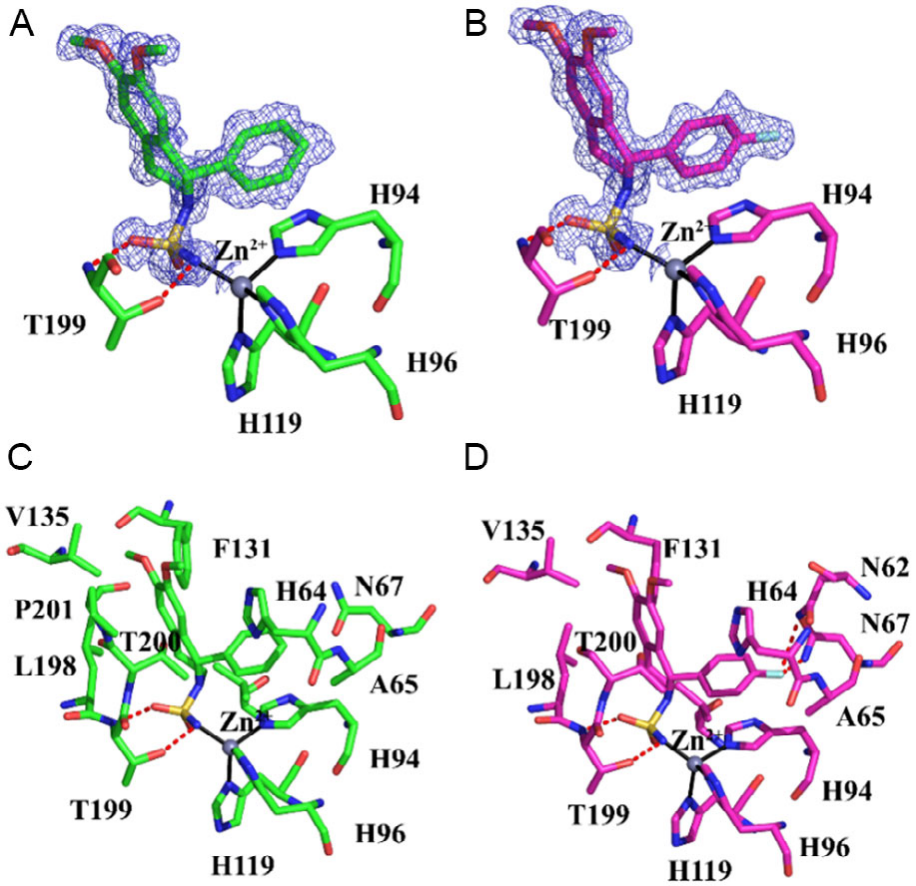
Active site view of hCA II/**1** (A) and hCA II/**2** (B) adducts showing the |2Fo‐Fc| electron density maps relative to the inhibitor molecule and contoured at 1 σ. Hydrophobic and polar interactions stabilizing the binding are represented in panels (C) and (D) for hCA II/**1** and hCA II/**2**, respectively. The Zn^2+^ ion coordination (solid lines) and hydrogen bond interactions (dashed lines) are also depicted.

Initially, we attempted to crystallize hCA II and hCA XII in complex with compounds **1–3**. While diffraction‐quality crystals were successfully obtained for hCA II in complex with inhibitors **1** and **2**, all crystallization attempts involving hCA XII were unsuccessful, preventing structural characterization of these complexes by X‐ray crystallography. As a result, the hCA XII binding mode to these compounds has been evaluated exclusively by computational modeling (vide infra). In the case of isoform II, crystals of the hCA II/**1** and hCA II/**2** adducts were obtained using the soaking technique, as described in the Experimental Section. Data collection and refinement of both adducts were carried out as reported in the Experimental Section (Table 2). Inspection of the initial |Fo − Fc| electron density maps for both structures immediately revealed the binding of the inhibitor molecule in the active site (Figure 3A,B). In both cases the sulfamide moiety adopts a binding mode to hCA II that is completely superimposable with that observed for other hCA/sulfamides complexes [22].

**TABLE 2:** Data collection and refinement statistics of inhibitors **1** and **2** bound to hCA II.

	hCA II/1	hCA II/2
**Cell parameters**		
Space group	P2_1_	P2_1_
Cell dimensions, Å, °	*a* = 42.2	*a* = 42.3
	*b* = 41.5	*b* = 41.4
	*c* = 72.0	*c* = 71.9
	*β* = 104.2	*β* = 104.2
Number of independent molecules	1	1
**Data collection statistics**		
Wavelength, Å	1.0	1.0
Resolution limits, Å	35.6–1.05	41.0–1.15
Total reflections	627 339	471 374
Unique reflections	104 567	82 177
Redundancy	6.0	5.7
Completeness, %	92.8 (84.1)	95.1 (89.4)
R‐merge^a^	0.065 (0.435)	0.086 (0.672)
Rmeas^b^	0.071 (0.496)	0.094 (0.773)
Rpim^c^	0.028 (0.235)	0.038 (0.372)
CC1/2^d^	0.998	0.999
<I>/<σ(I)>	20.9 (3.4)	22.0 (2.0)
**Refinement statistics**		
Resolution limits, Å	35.6–1.05	41.0–1.15
R‐work^e^, %	17.0	13.2
R‐free^e^, %	18.7	15.9
**r.m.s.d. from ideal geometry**		
Bond lengths, Å	0.011	0.014
Bond angles, °	1.7	1.9
Number of protein atoms	2073	2114
Number of inhibitor atoms	24	25
Number of water molecules	232	231
**Average B factor (Å^2^)**		
All atoms	12.94	14.08
Protein atoms	12.08	13.12
Inhibitor atoms	11.47	12.03
Waters	20.55	22.81
PDB accession code	30TA	30XX

a R‐merge = Σ_hkl_Σ_i_|I_i_(hkl)‐<I(hkl)>|/Σ_hkl_Σ_i_I_i_(hkl), where I_i_(hkl) is the intensity of an observation and <I(hkl)> is the mean value for its unique reflection; summations are over all reflections.

b Rmeas = Σ_hkl_{N(hkl)/[N(hkl)−1]}^1/2^ × Σ_i_|I_i_(hkl)‐<I(hkl)>|/Σ_hkl_Σ_i_I_i_(hkl).

c Rpim = Σ_hkl_{1/[N(hkl)−1]}^1/2^ × Σ_i_|I_i_(hkl)‐<I(hkl)>|/Σ_hkl_Σ_i_I_i_(hkl).

d CC1/2 = <I2>  − <I > 2/<I2>  − <I > 2  +  σ2ε (the value refers to the highest resolution shells).

e R‐work = Σhkl||Fo(hkl)|  −  |Fc(hkl)||/Σ_hkl_|Fo(hkl)| calculated for the working set of reflections. R‐free is calculated as for R‐work, but from data of the test set that was not used for refinement (Test Set Size (%) = 1.2% and 1.3% for hCA II/**1** and hCA II/**2**, respectively). Values in parentheses are referred to the highest resolution shell (1.07–1.05 Å and 1.17–1.15 Å for hCA II/**1** and hCA II/**2**, respectively).

In particular, the deprotonated NH^−^ group of the sulfamide binds the catalytic zinc ion in a tetrahedral geometry, acting as a hydrogen bond donor to the T199 OG1 atom. Concurrently, one of the sulfamide oxygen atoms serves as a hydrogen bond acceptor from the T199 backbone amide. In both structures, the 3,4‐dihydroisoquinoline skeleton is oriented toward the hydrophobic region of the active site cavity, where it forms strong hydrophobic contacts (<4.0 Å) with residues V121, F131, V135, L198, and P201, whereas the phenyl substituent is directed toward the hydrophilic region of the active site, where it interacts with the residues H64, A65, N67, H94, and T200 (Figure 3C,D). Interestingly, the introduction of a fluorine atom on the phenyl ring of compound **2** does not alter the overall orientation of the inhibitor within the enzyme active site; however, it contributes to the binding through weak polar interactions with the NH_2_ moieties of N62 and N67 side chains (Figure 3D). The high similarity in the binding modes of compounds **1** and **2** to hCA II is consistent with the very similar *K*_i_ inhibition values observed for these two molecules. Notably, although soaking experiments were carried out using a racemic mixture of compounds **1** and **2**, only the *S* enantiomer was observed in both structures, suggesting enantioselective binding to the enzyme.

To better understand the structural basis underlying the poor inhibitory properties of compounds **1** and **2** against hCA II, the crystallographic structures of their complexes with this isoform were superimposed onto the crystal structures of the same enzyme in complex with **AAZ** (PDB code 3HS4) [23] and with the analog 6,7‐dimethoxy‐1‐methyl‐3,4‐dihydroisoquinoline‐2(1*H*)‐sulfonamide **4** (PDB code 3PO6) [24], both of which act as significantly more potent hCA II inhibitors (*K*_i_ values of 12.1 and 87.3 nM, respectively). The chemical structure of compound **4** is reported in Figure S1.

**FIGURE 4:**
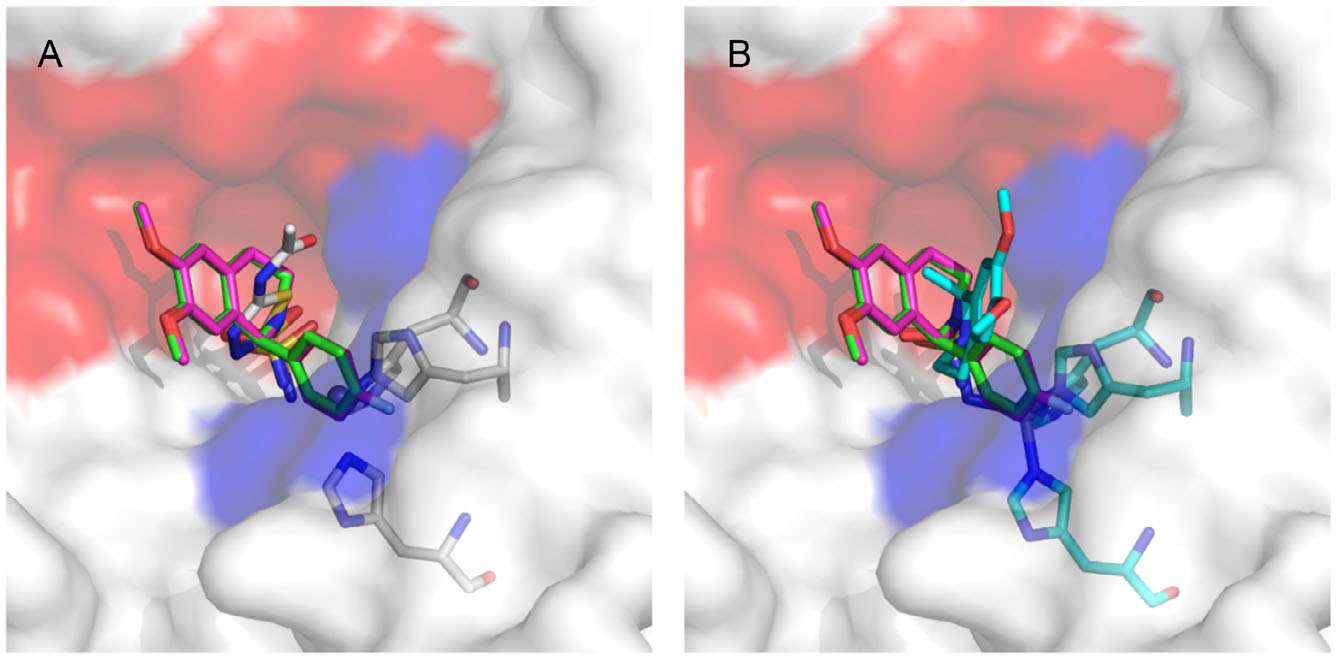
Superimposition of inhibitors (*S*)‐**1** (green) and (*S*)‐**2** (magenta) with AAZ (PDB code 3HS4, white) [23] (A) and 6,7‐dimethoxy‐1‐methyl‐3,4‐dihydroisoquinoline‐2(1*H*)‐sulfonamide **4** (PDB code 3PO6, cyan) [24] (B) when bound to the hCA II active site. In both panels hCA II is shown in surface representation, with the hydrophobic and hydrophilic regions colored in red and blue, respectively.

As shown in Figure 4A,B, although all four inhibitors share a common positioning of the zinc‐binding group, compounds **1** and **2** adopt a markedly different spatial orientation of the organic scaffold compared to **AAZ** and compound **4**. Indeed, the latter two molecules are in the central region of the active site cavity, whereas compounds **1** and **2**, being significantly larger, almost completely fill the cavity, with the dihydroisoquinoline ring and the C1 substituent oriented toward the hydrophobic and hydrophilic regions, respectively. As a consequence, the hydrophobic C1 aryl moiety is forced into a hydrophilic region of the cavity, a feature that likely underlies the reduced inhibitory activity of compounds **1** and **2** (see Table 1).

### 2.3 Development of an Ensemble Docking Protocol for hCA XII

Due to the lack of available crystallographic data for molecules **1** and **2** bound to hCA XII (cfr supra), we applied a computational approach to elucidate the molecular interactions of these compounds with hCA XII and to characterize the protein–ligand contacts responsible for their selectivity over hCA II as displayed in Table 1.

**FIGURE 5:**
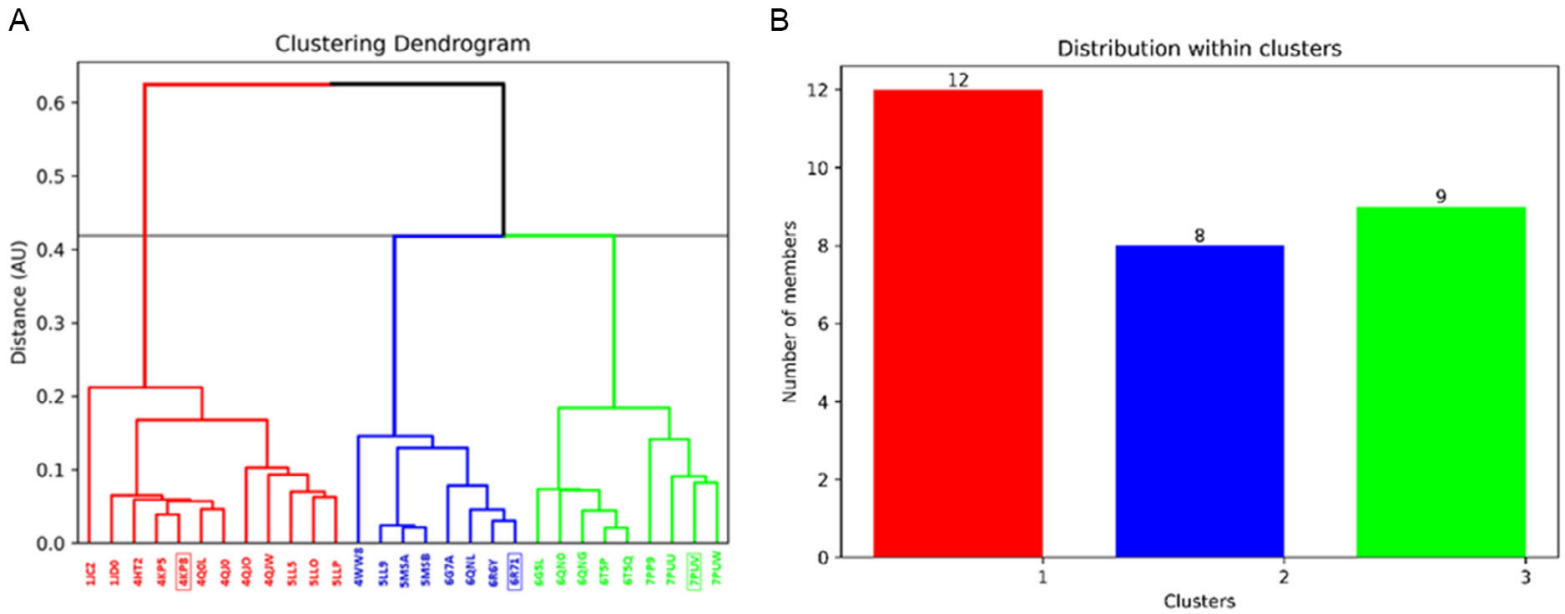
(A) Dendrogram generated by the Matplotlib package included in TTClust for hCA XII, showing the ward linkage matrix of the clustering run. The *X* axis indicated the PDB structures used, while the *Y* axis showed the distance at which distinct members were merged into the same cluster. The gray horizontal line represented the distance cut‐off chosen by the auto‐clustering algorithm to define the final number of generated clusters. On the *X* axis, representative frames were highlighted by the colored squares. (B) Histograms showing the population of each cluster.

To account for protein flexibility without compromising the computational speed, we employed an ensemble docking strategy based on multiple hCA XII crystal structures cocrystallized with different inhibitors available in the RCSB Protein Data Bank. In the Experimental Section we carefully described the protocol used to generate the ensemble model. Briefly, the workflow included the following steps: a) selection and preparation of the protein structures; b) clustering of PDB structures; and c) validation of the docking protocol. In detail, several hCA XII PDB structures (accession date March 2nd, 2023) including both apo forms and adducts with substrate or inhibitors were collected. Protein selection was carried out according to the nature of the ligands as detailed in Experimental Section. Then, using representative conformations of the protein obtained via root mean square deviation (RMSD) hierarchical clustering, an in–depth validation of the docking procedure was performed (Figure 5). Agglomerative hierarchical clustering, performed using TTClust, produced three representative conformations of hCA XII (Figure 5). These set of structures were subsequently used in the docking studies, using Glide Virtual Screening workflow as described in Experimental Section. Validation of the ensemble docking protocol was reported in the Supporting Material for ligands cocrystallized with hCA XII (see Table S1).

During redocking and cross‐docking experiments, the above‐described ensemble docking method was able to reproduce the native conformation of 24 out of 28 (85.17%) ligands cocrystallized with hCA XI with an RMSD value ≤2.6 Å. Additionally, 45.83% of this subset (11 out of 24 ligands for hCA XII) were obtained by docking the ligand into the representative structure of the cluster that included the native protein, thus supporting the employment of protein clustering procedure prior to performing ensemble docking (see Table S2).

Once validated the protocol, the representative protein structures obtained via RMSD hierarchical clustering were employed for modeling studies involving compounds **1** and **2.** The binding modes were visualized with PyMOL (https://pymol.org). Despite the inhibitory activity was measured for the racemates of compounds **1** and **2**, we focused our attention on the (*S*)‐enantiomers, as crystallographic analysis showed that only this enantiomer is present in the hCA II active site (see previous section).

**FIGURE 6:**
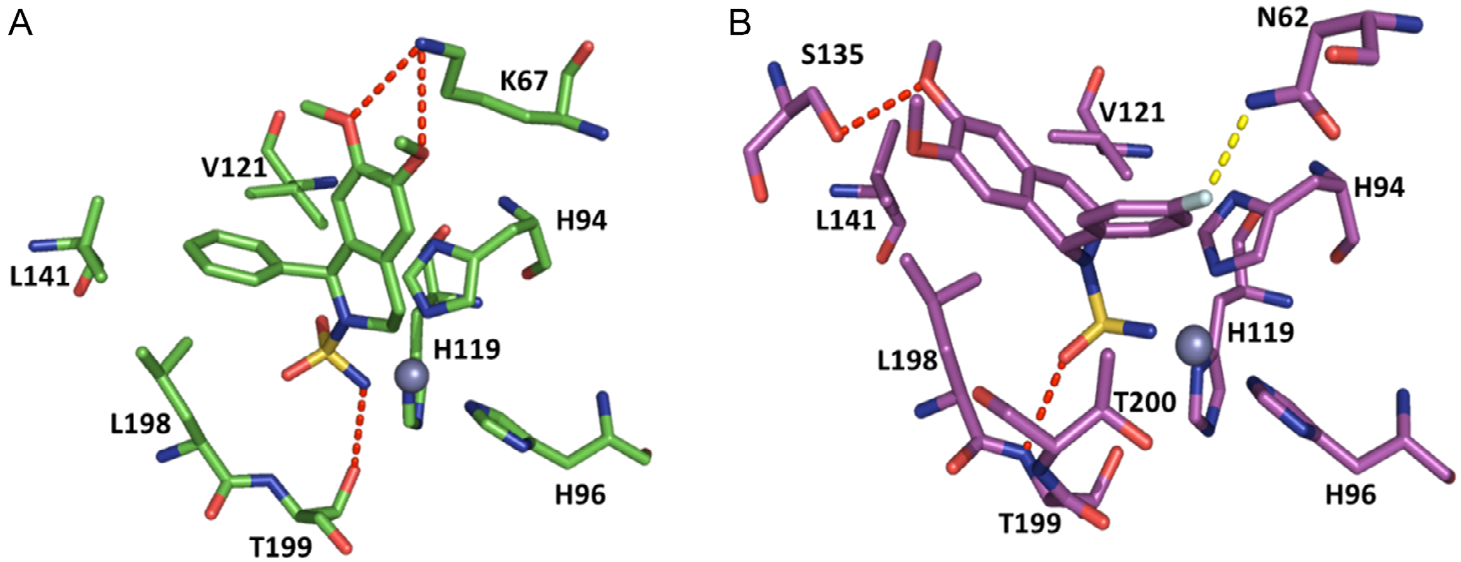
Predicted binding mode of (*S*)–enantiomer of inhibitor **1** (A, green sticks) and **2** (B, purple sticks) in the hCA XII catalytic cavity. Polar interactions are depicted as dashed red lines or dashed yellow lines (contacts through fluorine atom).

As shown in Figure 6, in both hCA XII/inhibitor docked complexes, the sulfamide moiety was anchored to the catalytic site through canonical interactions with the catalytic zinc ion, whereas the isoquinoline core adopted markedly different orientations in the two compounds.

For the inhibitor (*S*)‐**1** (Figure 6A, green sticks) the C‐1 aryl substituent occupied the hydrophobic pocket defined by V121, L141, and L198 near the entrance of the active site, whereas the two methoxy substituents of the isoquinoline ring engaged through their oxygen atoms in a bidentate hydrogen‐bond interaction with the nonconserved K67 residue. In the case of (*S*)‐**2** (Figure 6B, purple sticks), the isoquinoline core structure was oriented toward the hydrophobic region, with one of the methoxy groups interacting with the side chain of S135, a distinctive residue of hCA XII. In this case the C‐1 aryl group participates in a hydrophobic interaction with residue T200. Finally, the anchoring of (*S*)‐**2** to hCA XII cavity is reinforced by a polar interaction between the 4‐fluorine atom and residue N62. This interaction could be responsible for stabilizing the different orientation of the C‐1 aryl ring of compound **2** compared to compound **1**.

**FIGURE 7:**
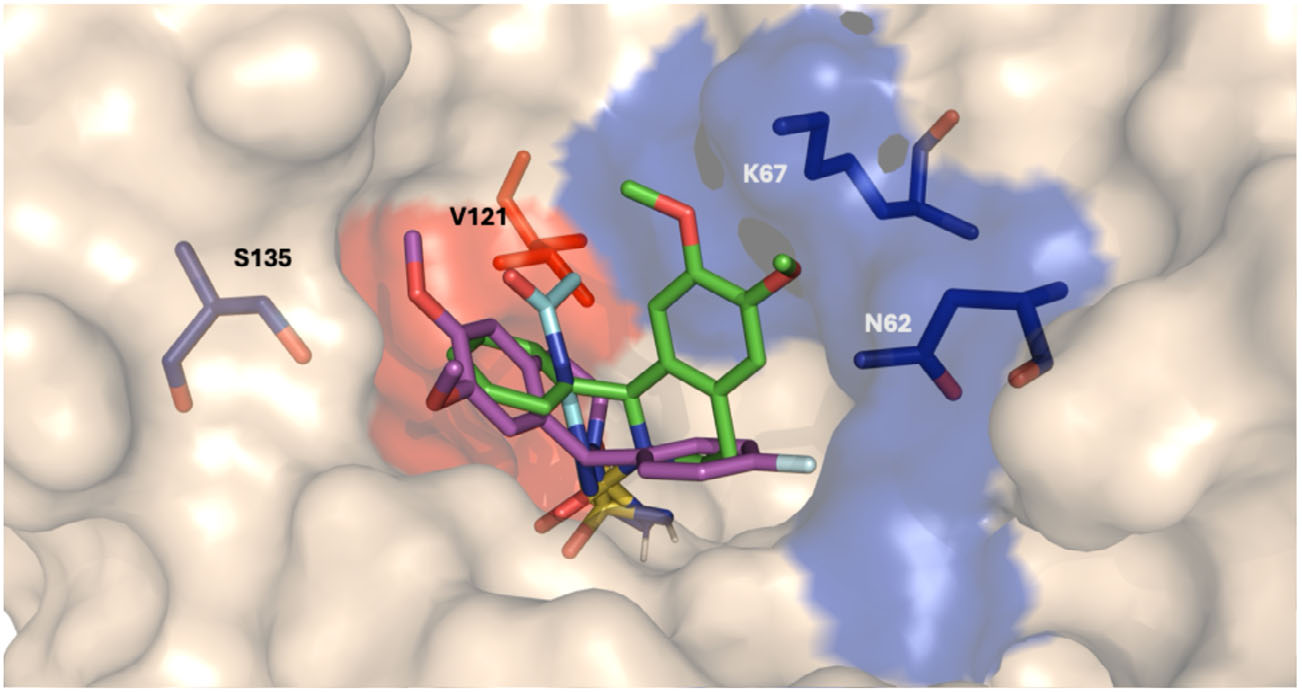
Superimposition of predicted poses of ligands **1** and **2** within the hCA XII active site with AAZ bound to the same enzyme as determined by X‐ray crystallography (PDB code 1JD0). The hCA XII active site is shown in surface representation with the hydrophobic region shown in blue, and the hydrophilic region in red. Key residues are depicted as sticks.

To gain further insight into the binding mode of compounds **1** and **2** toward hCA XII, their predicted binding poses within the enzyme active site were compared with the binding mode of AAZ in the same enzyme [25]. Notably, AAZ exhibits comparable Ki values (see Table 1) while displaying a different selectivity profile toward hCA II. As depicted in Figure 7, the three ligands share a conserved positioning of the zinc‐binding group. However, whereas the acetamido‐thiadiazolyl tail of AAZ is oriented toward the hydrophobic wall, forming contacts with V121 via its methyl moiety, compounds **1** and **2** establish a broader network of favorable interactions involving both hydrophilic and hydrophobic regions lining the active site cavity.

For all studied complexes (hCA II and hCA XII with compounds **1, 2** and AAZ), binding energy values were evaluated using molecular mechanics/generalized born surface area calculations, showing a good agreement with the inhibitory activities reported in Table 1. Specifically, the data collected in Table 3 evidenced that compounds **1** and **2** exhibit more negative binding energy values when docked into hCA XII compared to those calculated for the same inhibitors in complex with hCA II.

**TABLE 3:** Binding energy values obtained from MM‐GBSA calculations of **1**, **2**, and AAZ on hCA II and hCA XII.

	MM–GBSA	ΔG binding energy
Entry	hCA II	hCA XII
(*S*)‐**1**	−5.74^a^	−20.5^b^
(*S*)‐**2**	−8.62^a^	−24.28^b^
AAZ	−22.81^a^	−22.13^a^

a X‐ray complexes PDB 3HS4 and 1JD0 AAZ bound to hCA II and hCA XII, respectively.

b Docking complexes.

The hypothetical binding modes of the (*R*)–enantiomers of compounds **1** and **2** to hCA XII were also investigated, revealing that both (*R*)–**1** and (*R*)–**2** can satisfactorily occupy the enzyme active site and establish favorable interactions with key residues (see Figure S8). This behavior may account for the remarkable hCA XII inhibitory activity observed for the racemic mixtures of **1** and **2**.

### 2.4 MD Simulations on hCA II and XII

**FIGURE 8:**
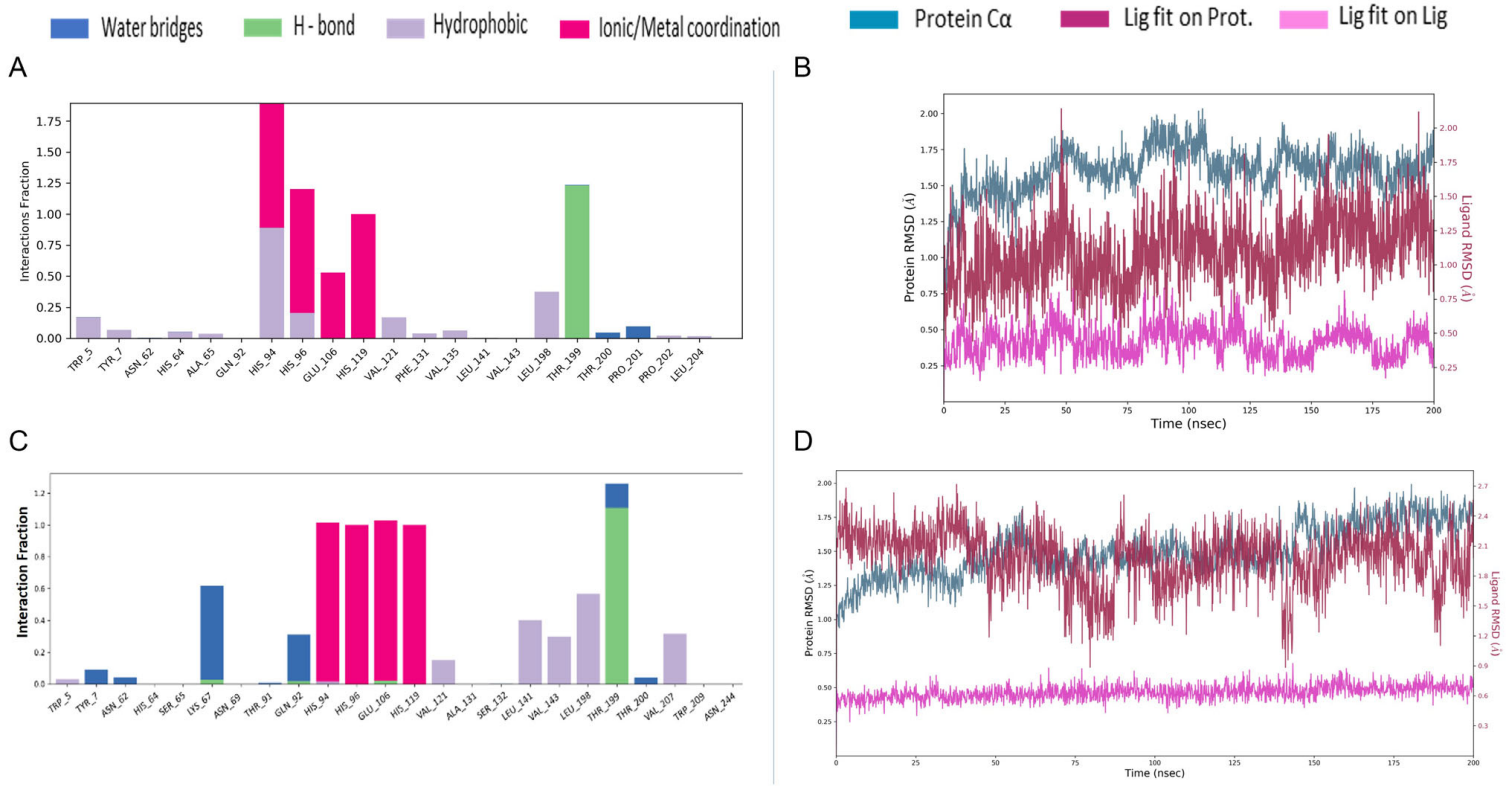
Desmond Simulation interaction diagram (SID) of compound (*S*)–**1** with hCA II, as observed during the 200 ns MD simulation based on the crystallographic structure (A,B) and with hCA XII, as observed during the 200 ns MD simulation on the docking complex (C,D). The protein–ligand contact bar plots (left), report the interacting residues on the *x* axis and the interaction frequency, expressed as fraction, on the *y* axis. The type of interaction established by each residue is indicated according to the color scheme on the top. Values with a fraction exceeding 0.1 (100%) occur when multiple type of contacts are formed by the same residue with the ligand or the metals ion, or when the same interaction involves multiple ligand atoms. RMSD plots along the MD simulations (right), show the protein Cα and ligand RMSD values (Å) along the simulation, calculated following the alignment scheme indicated by the color coding at the top.

**FIGURE 9:**
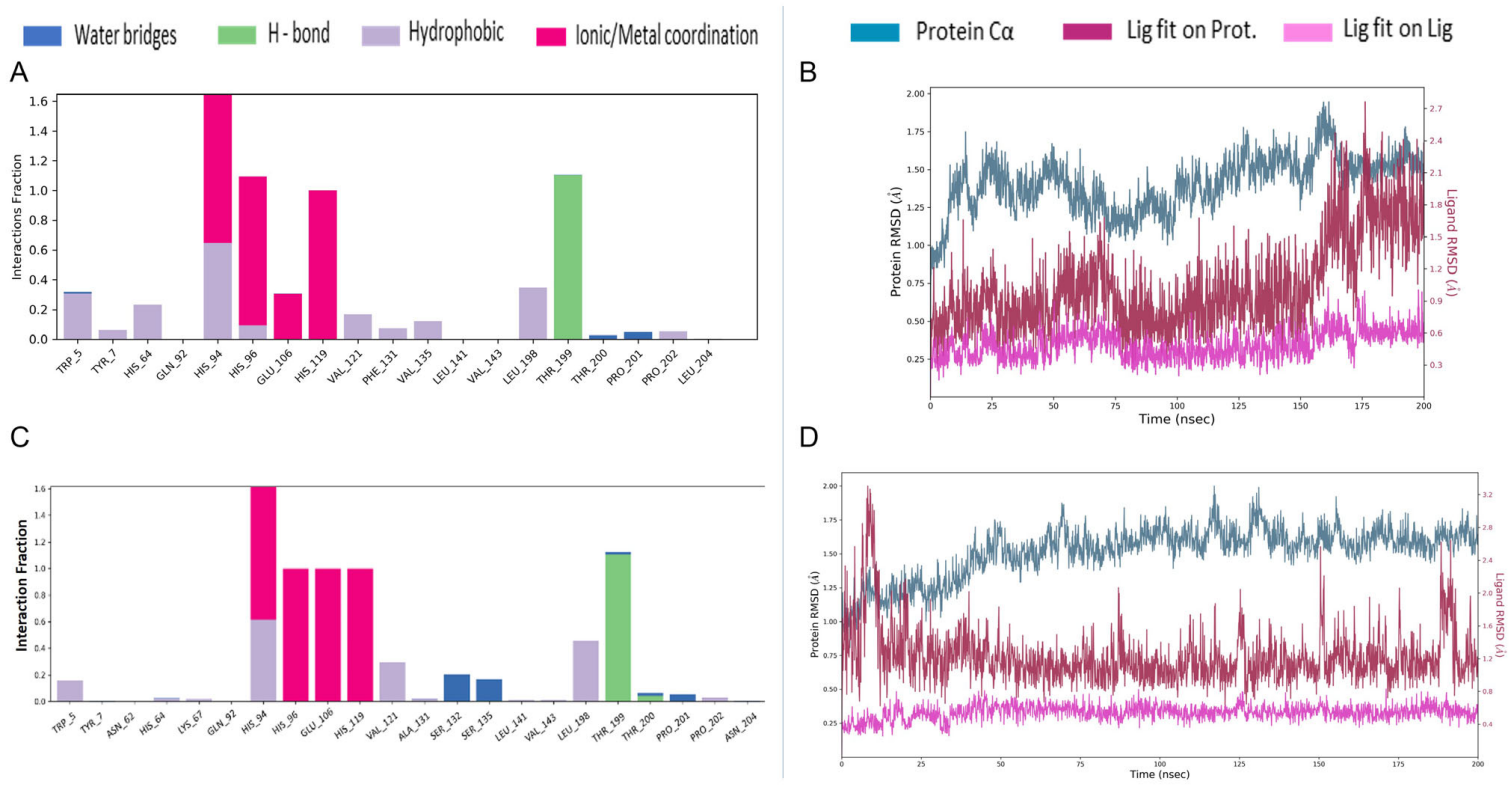
Desmond simulation interaction diagram (SID) of compound (S) – **2** with hCA II as observed during the 200 ns MD simulation based on the crystallographic structure (A,B) and with hCA XII as observed during the 200 ns MD simulation on the docking complex (C,D). The protein– ligand contact bar plots (left), report the interacting residues on the *x* axis and the interaction frequency, expressed as fraction, on the *y* axis. The type of interaction established by each residue is indicated according to the color scheme at the top. Values with a fraction exceeding 0.1 (100%) occur when multiple type of contacts are formed by the same residue with the ligand or the metals ion, or when the same interaction involves multiple ligand atoms. RMSD plots along the MD simulations (right), show the protein Cα and ligand RMSD values (Å) along the simulation, calculated following the alignment scheme indicated by the color coding at the top.

To further reinforce our model for the selective binding of inhibitors **1** and **2** to hCA XII and to gain deeper insights into the protein/ligand interactions, 200 ns Molecular dynamics (MD) simulations were performed using the Desmond package on the docking complexes with hCA XII as well as on the crystallographic structures of the hCA II/inhibitor complexes. Comparative analysis of the MD simulations was carried out to provide insights into potential differences in complex stability, expressed in terms of ligand RMSD values or interaction occurrence throughout the simulations. The Figures 8 and 9 display the results of the MD simulations for studied inhibitors (*S*)‐**1**, (*S*)‐**2**, as detailed below.

By analyzing the MD simulations, it was observed that the inhibitors (*S*)‐**1** and (*S*)‐**2** stably coordinated the zinc ion and were hydrogen bound to T199 throughout the simulation within the active site of hCA II (Figures 8A and 9A) and hCA XII (Figures 8C and 9C). Moreover, they preserved the hydrogen bonding networks (mainly mediated by water bridges) and the hydrophobic interactions at the entrance of the hCA XII binding site (Figures 8C and 9C), as detected in the minimized docked poses (see Figure 5). In the case of hCA II, the only observed interactions involved H94; no hydrogen bonds stabilized the binding of the two compounds or justified the positioning of the aryl moiety toward the hydrophilic region of the enzyme active site (Figures 8A and 9A).

This behavior was confirmed by the ligand RMSD values, which showed a very irregular slope trend, thus reflecting changes in the position and conformation of the ligands (See Lig fit on Protein and Lig fit on Lig of Figures 8B and 9B).

Overall, compounds **1** and **2** displayed markedly higher stability in complex with hCA XII during the MD simulations compared to hCA II, as evidenced by more stable RMSD profiles and distinct interaction profiles. These computational findings provided a rationale for the selective inhibition of hCA XII by compounds **1** and **2**.

## 3 Conclusion

This study reports experimental and computational investigations aimed at elucidating the molecular determinants responsible for the selective binding of 1‐aryl‐6,7‐dimethoxy‐3,4‐dihydroisoquinoline‐2(1*H*)‐sulfonamides **1–2** toward hCA XII over hCA II. In particular, the computational analysis consisted of ensemble docking, binding free energy calculations, and MD simulations on hCA XII; the computational outcomes were integrated with crystallographic studies performed on the hCA II isoform. The results indicated that the investigated compounds effectively occupy the hCA XII active site through profitable interactions underlying the unexpected high isoform selectivity, which could be exploited for development of further agents for cancer therapy.

## 4 Experimental Section

### 4.1 Chemistry

1‐Aryl‐6,7‐dimethoxy‐3,4‐dihydroisoquinoline‐2(1*H*)‐sulfonamides (**1–3**) have been obtained as racemate by a synthetic procedure that was described in our previous paper [14] and they were available in‐stock to perform biochemical assays as well as crystallographic studies. In detail, the compounds were collected from our repository CHIME containing 1,683 compounds (CHIME2024). The three selected 1‐aryl‐6,7‐dimethoxy‐3,4‐dihydroisoquinoline‐2(1*H*)‐sulfonamides (**1–3**) possessed >95% purity and were characterized by ^1^H and ^13^C NMR spectroscopy on Varian Gemini 500 (Palo Alto, CA, USA) in DMSO *d*_6_. Melting points were recorded on Buchi B‐545 (BUCHI Labortechnik AG, Flawil, Switzerland). The purity and structural determination was carried out before to perform biochemical assays against hCAs.

#### 4.1.1 6,7‐Dimethoxy‐1‐Phenyl‐3,4‐Dihydroisoquinoline‐2(1H)‐Sulfonamide (1, CAS Number: 1 161 432‐33−2)

Off‐white powder soluble in DMSO; m.p. 186°C–188°C; ^1^H NMR (500 MHz, DMSO‐*d*_6_, ppm): δ = 2.56–2.59 (m, 1H, CH_2_), 3.01–3.10 (m, 2H, CH_2_), 3.56–3.58 (m, 1H, CH_2_), 3.61 (s, 3H, CH_3_), 3.74 (s, 3H, CH_3_), 5.84 (s, 1H, CH), 6.57 (s, 1H, Ar‐H), 6.77 (s, 1H, Ar‐H), 6.84 (s, 2H, ‐SO_2_NH_2_), 7.17–7.30 (m, 5H, Ar‐H); ^13^C NMR (126 MHz, DMSO‐*d*_6_): δ = 26.3, 31.1, 55.9, 56.0, 58.4, 112.1, 112.3, 126.6, 127.1, 127.5, 128.5 128.7, 143.3, 147.4, 148.2; elemental analysis calcd (%) for C_17_H_20_N_2_O_4_S: C 58.60, H 5.79, N 8.04; found: 58.63, H 5.76, N 8.01.

#### 4.1.2 1‐(4‐Fluorophenyl)−6,7‐Dimethoxy‐3,4‐Dihydroisoquinoline‐2(1H)‐Sulfonamide (2, CAS Number: 1 161 432‐38−7)

Off‐white powder soluble in DMSO; m.p. 194°C–196 °C. ^1^H NMR (500 MHz, DMSO‐*d*_6_, ppm): δ = 2.56–2.59 (m, 1H, CH_2_), 3.00–3.06 (m, 2H, CH_2_), 3.58–3.60 (m, 1H, CH_2_), 3.61 (s, 3H, CH_3_), 3.74 (s, 3H, CH_3_), 5.84 (s, 1H, CH), 6.58 (s, 1H, Ar‐H), 6.78 (s, 1H, Ar‐H), 6.87 (s, 2H, SO_2_NH_2_), 7.09–7.21 (m, 4H, Ar‐H); ^13^C NMR (126 MHz, DMSO‐*d*_6_): δ = 26.3, 31.1, 55.9, 56.0, 57.7, 112.0, 112.3, 115.2 (d, ^2^ *J*_C‐F_ = 21 Hz), 126.5, 127.0, 130.6 (d, ^3^ *J*_C‐F_ = 7.6 Hz), 139.6 (d, *J* ^4^_C‐F_ = 2.9 Hz), 147.5, 148.3, 161.7 (d, ^1^ *J*_C‐F_ = 244 Hz); elemental analysis calcd (%) for C_17_H_19_FN_2_O_4_S: C 55.73, H 5.23, N 7.65; found: 55.71, H 5.20, N 7.68.

#### 4.1.3 *1‐*(*4‐Aminophenyl*)*‐6*,*7‐DImethoxy‐3*,*4‐DIhydroisoquinoline‐2*(*1H*)*‐Sulfonamide* (*3*, *CAS Number: 1 161 432‐43‐4*)

Off‐white powder soluble in DMSO; m.p. 197°C–199°C. ^1^H NMR (500 MHz, DMSO‐*d*_6_, ppm): δ = 2.53–2.55 (m, 1H, CH_2_), 2.98–3.03 (m, 2H, CH_2_), 3.55–3.57 (m, 1H, CH_2_), 3.60 (s, 3H, CH_3_), 3.73 (s, 3H, CH_3_), 5.0 (bs, 2H, Ar‐NH_2_), 5.68 (s, 1H, CH), 6.43–6.47 (m, 3H, Ar‐H), 6.67 (bs, 2H, SO_2_NH_2_), 6.73 (s, 1H, Ar‐H), 6.77–6.79 (m, 2H, Ar‐H);^13^C NMR (126 MHz, DMSO‐*d*_6_): δ = 26.3, 39.1 55.8, 55.9, 58.2, 111.9, 112.1, 113.7, 126.8, 127.5, 127.4, 129.6, 130.0, 147.22, 147.9, 148.2; elemental analysis calcd (%) for C_17_H_21_N_3_O_4_S: C 56.18, H 5.82, N 11.56; found: 56.21, H 5.79, N 11.54.

### 4.2 Carbonic Anhydrase Assay

The CA inhibition has been measured by a stopped‐flow method monitoring CO_2_ hydration, as reported earlier [10, 21].

### 4.3 Crystallographic Analysis

Crystals of hCA II/**1** and hCA II/**2** adducts were obtained using the soaking technique [22]. In particular, hCA II native crystals were prepared at room temperature by vapor diffusion hanging drop method by mixing 2 µL of protein (10 mg/ml in 0.02 M Tris‐HCl pH 8.0) with an equal volume of precipitant solutions containing 1.3 M sodium citrate and 0.1 M Tris‐HCl, pH 8.5. The drops were subsequently allowed to equilibrate against a 1 mL reservoir of the identical precipitant solution. A few native enzyme crystals were then transferred into a drop of freshly prepared precipitant solution, supplemented with 20 mM inhibitor and 15% (v/v) glycerol to serve as a cryoprotectant. These crystals were kept in the soaking solution overnight and then flash cooled in liquid nitrogen. Complete X‐ray data set were collected on the XRD2 beamline at the Elettra synchrotron‐radiation source, Trieste, Italy using a Dectris PILATUS 6 M detector at 100 K [26]. Diffraction data were processed and scaled with HKL2000 (HKL Research) to a maximum resolution of 1.05 Å for hCA II/**1** and 1.15 Å for hCA II/**2** [27]. Data collection statistics are reported in Table 2. Phasing and refinement of both hCA II complexes were carried out with REFMAC5 [28] using as starting model the atomic coordinates of the native enzyme with waters removed (PDB entry 1CA2) [29]. Unambiguous electron density corresponding to the two inhibitors was visible in the difference map following a single round of refinement. After an initial refinement limited to the enzyme structure, the inhibitor model was easily built for both hCA II adducts and introduced into the atomic coordinate sets for further refinement. Restraints on inhibitor bond angles and distances were taken from the Cambridge Structural Database [30], whereas standard restraints were used on protein bond angles and distances throughout refinement. Several rounds of manual rebuilding were performed using the program O [31] yielding final crystallographic R‐factor and R‐free values of 0.170 and 0.187 for hCA II/**1** and 0.132 and 0.159 for hCA II/**2**. The stereochemical quality and geometric restraints of the final model were validated with PROCHECK [32] and WHATCHECK [33] programs. The refinement statistics of the final model are summarized in Table 2. Coordinates and structure factors have been deposited in the Protein Data Bank (accession codes 30TA and 30XX).

### 4.4 Computational Studies

#### 4.4.1 Selection and Preparation of Protein Complexes

From RCSB Protein Data Bank we collected three‐dimensional structures of hCA XII isoforms of hCA. The selection criterion was the collection of the apo‐structures or complexes containing sulfonamides, sulfamides, and sulfamates, thus restricting the ensemble to 41 PDBs for hCA XII. Then, 12 hCA XII structures were discarded as displaying a chimeric sequence so that we reduced this set to 29 structures (see Table S1). Subsequently, we selected the chain A of each complex; then, we aligned the sequences by multiple sequence alignment using the Multiple sequence viewer in Maestro and the superimposition of the backbones was performed using as reference the hCA XII in complex with acetate (PDB 1JCZ). Taking as reference the PDB with the shortest sequence, the N and C termini were truncated. Next, each complex was processed by using the protein preparation workflow tool implemented in Maestro [34] as detailed below. In “review and modify” panel, we removed alternates with the lowest occupancy for each residue and detected cocrystalized ligand and cofactors. The “preprocess” step was carried out to achieve the following tasks: (a) assign bond order by using the CCD database; (b) add/replace hydrogens; (c) create zero‐order bonds to metals; (d) convert selenomethionine to methionine residue; (e) fill in missing loops (using Prime); (f) generate het states (with Epik) setting a pH range of 7.4 +/−2.0; (g) fill in missing side chains (with Prime); and (h) cap termini. The “diagnose and analyze” step allowed us to check issues such as overlaps, missing atoms, and wrong valences and to check the protonation states assigned by Epik in the previous step to the ligands and to the histidine residues; in the case of detection of multiple states for the ligand, we kept the first one for which the sulfonamide group displayed the nitrogen atom in deprotonated form, able to coordinate the zinc ion; histidine protonation states were also adjusted in some cases to have the same protonation state in all the protein, generating the same topology in each structure; no further adjustments were needed in all the hCA XII complexes, and the ligands were extracted from the complexes.

#### 4.4.2 PDB Clustering

After the extraction of the ligands from the corresponding complexes of hCA XII, we sampled a unique centroid from the superimposed ligand heavy atoms using Discovery Studio. Then, by means of VEGA ZZ software, we renumbered the residues of each protein structure to generate structures possessing the same topology; in detail, we started from number 3 for hCA XII; then, we identified the residues at 10 Å from the centroid coordinates present in the PDB used as reference for the initial superimposition (PDB 1JCZ for hCA XII; the selected residues are listed below: (W5; Y7; N65; H67; S68; K70; L71; T89; Q90; L91; H92; L93; H94; E105; H118; I119; V121; H122; Y123; A131; S132; S135; L141; A142; V143; S197; L198; T199; T200; P201; P202; C203; N204; T206; V207; and W209). Finally, we merged all the PDB structures in VMD [35] software as a unique trajectory, saved as DCD file, and we performed RMSD clustering analysis using TTClust tool [36]. The RMSD matrix was created considering backbone and sidechain atoms of the residues just listed above. Auto‐clustering was employed for the calculation, and Ward was set as clustering method (default). Frame was set as unit for the timed barplot. The representative frame of each cluster was then selected for our docking studies.

**Table:** 

PDB codes of selected hCA XII structures	
Cluster 1	4KP8
Cluster 2	6R71
Cluster 3	7PUV

#### 4.4.3 Ensemble Docking Setting

By using the default settings of the Glide Receptor grid generation tool, we generated a grid based on the cocrystallized ligand for each of the above‐listed PDB structures. All the ligands were initially sketched as mol files with ChemDraw, the structures were merged into one SDF file, and then minimized 3D coordinates were created using LigPrep; the chirality was retained when specified; otherwise, we created a maximum of 32 stereoisomers, generating possible states (with Epik) at a target pH of 7.0 +/−2.0 and adding metal coordination states. For each ligand the states with lowest metal state penalty and the sulfonamide nitrogen deprotonated were selected for our docking studies. Then, the following were applied for ensemble docking using the Glide Virtual Screening workflow (Vsw) [37]: a) dock using Glide SP, b) dock flexibly, c) generate a maximum of 20 poses per ligand, d) postminimize docking poses, e) After docking, keep 100% of the docked ligands, and f) retain all good scoring states. In the case of docking into multiple receptor grids using only the SP protocol, the Glide Vsw returns by default the pose with the best (the most negative) Glide SP score and the ensemble receptor associated with the selected pose. The fine‐tuned settings reported above belongs to a validation based on redocking and cross‐docking experiments conducted evaluating the ability to reproduce the native conformation of the ligands cocrystallized on the 29 protein structures clustered; the process employed the representative conformations that have been obtained by the clustering procedure. From the validation set of hCA XII, we discarded just the PDB 1JCZ, as it represents the apo‐protein. We considered the method successful if more than the 50% of the selected docking poses reproduce the X‐ray conformation with an RMSD value ≤2.6 Å. (See validation report in Table 6A and 6B of Result and discussion).

#### 4.4.4 MM—GBSA Binding Energy Calculation

Docking complexes obtained from the Vsw protocol were first refined using the Prime refine complexes panel, performing a local optimization of the residues at 5 Å from the ligand centroid and employing an implicit VSFG water model and OPLS4 forcefield on Schrödinger Suite. For all complexes, apart from the residue flexibility, which were kept fix, the same water model and forcefield were set during binding energy calculation. Apart from collecting the Δ*G* bind values, the results were saved as well as PDB and employed for interaction analysis and binding mode comparison using LigandScout and Pymol software, respectively.

#### 4.4.5 MD Simulation on hCA II and XII

The refined complexes obtained from the MM – GBSA binding energy calculation and the hCA II X‐ray structures of compound **1** and **2** were submitted to the “Protein Preparation Workflow” as described in the “Selection and preparation of protein complexes” section, additionally refining the polar hydrogen orientation through the “Optimize hydrogen‐bonds” panel, although keeping fix the protomers and tautomers of H64(proton‐shuttling histidine) and H94, H96, and H119 (Zinc‐coordinating histidines). MD simulations were performed on Desmond software (2021). An explicit 10 × 10 x 10 orthorombic TIP4PEW water box was generated for each complex submitted to the MD simulation. Na^+^ and Cl^−^ counterion were added after charge calculation, as well as an additional 0.15 M salt buffer of the same ions. OPLS4 forcefield were employed also in this case. A 200 ns MD production phase using NPT ensemble with Martyna–Tobias–Klein barostat and Nose–Hoover chain thermostat was performed on the systems just prepared. The default Desmond protocol was used for equilibration and relaxation of the systems. Atom coordinates during production step were collected each 100 ps obtaining ≈2000 frames per simulation.

## Author Contributions

All authors have read and agreed to the published version of the manuscript.

## Funding

This study was supported by Ministero dell’Istruzione, dell’Università e della Ricerca (PRIN 201744BN5).

## Conflicts of Interest

The authors declare no conflicts of interest.

## Supporting information

Supplementary Material

## Data Availability

The data that support the findings of this study are available in the supplementary material of this article as Supporting Information.
