# Supplementary material for: 1‐Aryl‐6,7‐Dimethoxy‐3,4‐Dihydroisoquinoline‐2(1H)‐Sulfonamides as hCA XII Selective Inhibitors: Experimental and Theoretical Studies to Interrogate the Isoform Selectivity
Source: ChemMedChem. 2026 Jul 23;21(14):e70390. doi: 10.1002/cmdc.70390 (PMC13395569; doi:10.1002/cmdc.70390)
Supplement: Supplementary file 1 — Supplementary Material [file CMDC-21-e70390-s001.pdf]

SUPPORTING INFORMATION

1-Aryl-6,7-dimethoxy-3,4-dihydroisoquinoline-2(1*H*)-sulfonamides as hCA XII selective inhibitors: experimental and theoretical studies to interrogate the isoform selectivity

Federico Ricci,[a] Anna Di Fiore,[b] Andrea Angeli,[c] Laura De Luca,[a] Francesca Mancuso,[a] Davide Esposito,[b] Giuseppina De Simone,[b] Claudiu T. Supuran,[c] and Rosaria Gitto,*[a]

[a]University of Messina, CHIBIOFARAM Department, I-98168, Messina Italy

[b]CNR Institute of Biostructures and Bioimaging, I-80131, Napoli, Italy

[c]University of Florence, NEUROFARBA Department, I-50019, Sesto Fiorentino (FI) Italy

*corresponding author: Rosaria Gitto rgitto@unime.it

Table of contents:

Figures S1-S6: ^{1}H-NMR and ^{13}C-NMR Spectral data for compounds 1-3 page S2

Figure S7: Chemical structure of 6,7-dimethoxy-1-methyl-3,4-dihydroisoquinoline-2(1*H*)-sulfonamide (**4**) page S8

Table S1. *K*i values of compounds **1-3** againsts hCA I, VII, IX and XIV. Page S9

Table S2. Validation report of the ensemble docking protocol to predict hCA XII poses page S10

Figure S8. Predicted binding mode of derivative (*R*)-1 and (*R*)-2 in the hCA XII catalytic cavity page S11

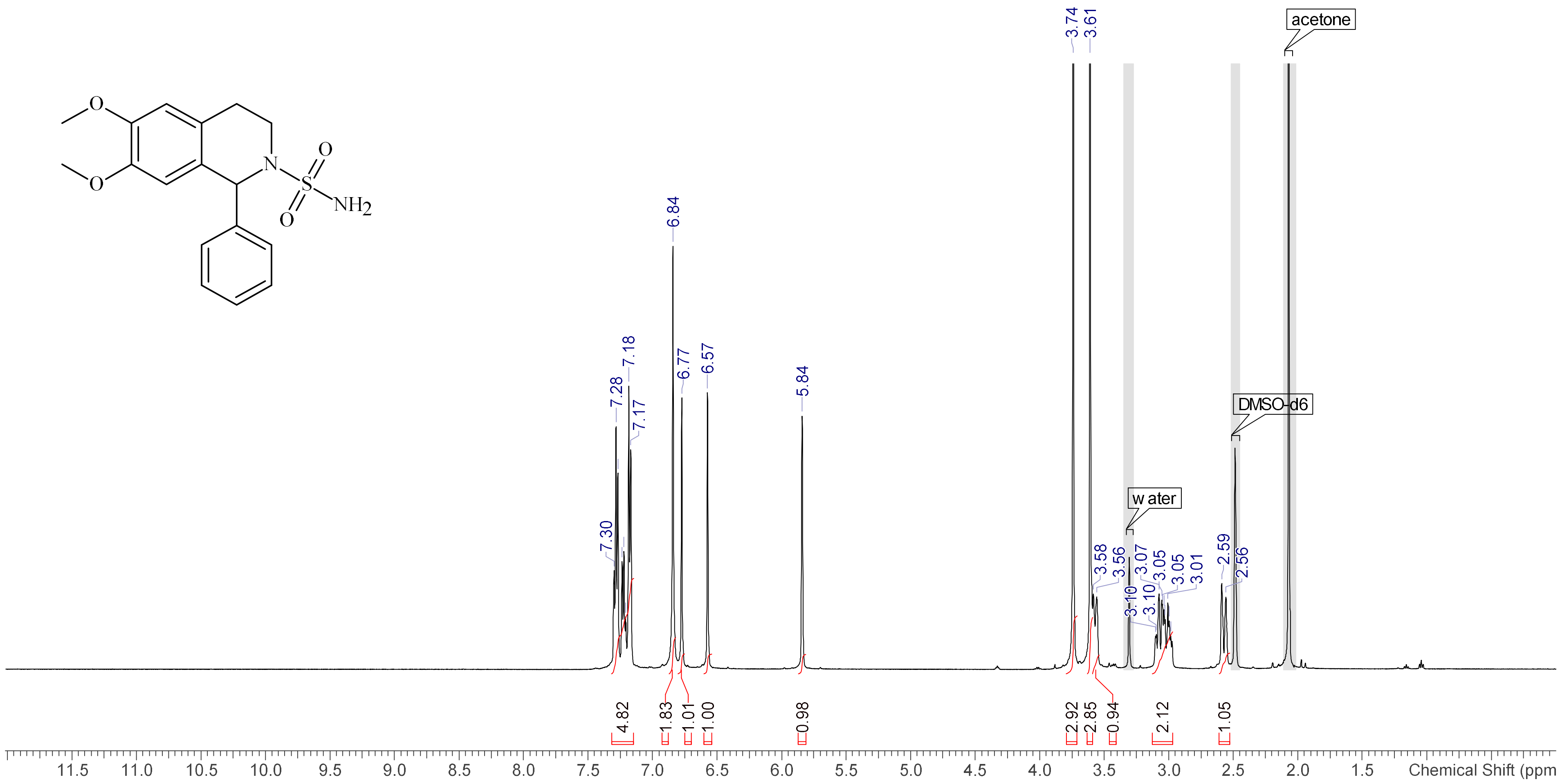

Figure S1: ^{1}H-NMR (DMSO-d_6) spectrum for 6,7-dimethoxy-1-phenyl-3,4-dihydroisoquinoline-2(1*H*)-sulfonamide (**1**)

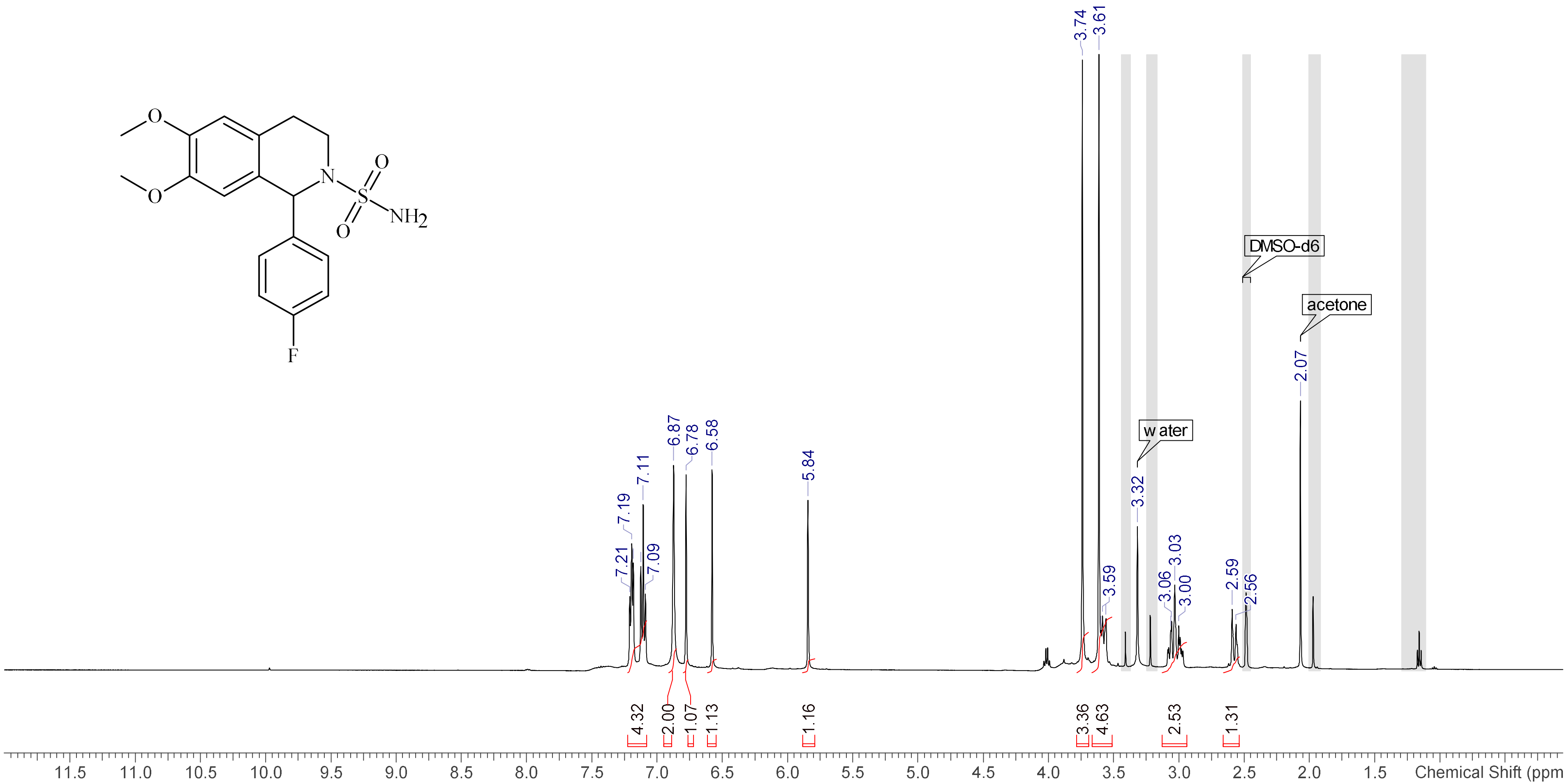

Figure S2: ^{1}H-NMR (DMSO-*d*$_6$) spectrum for 1-(4-fluorophenyl)-6,7-dimethoxy-3,4-dihydroisoquinoline-2(1*H*)-sulfonamide (**2**)

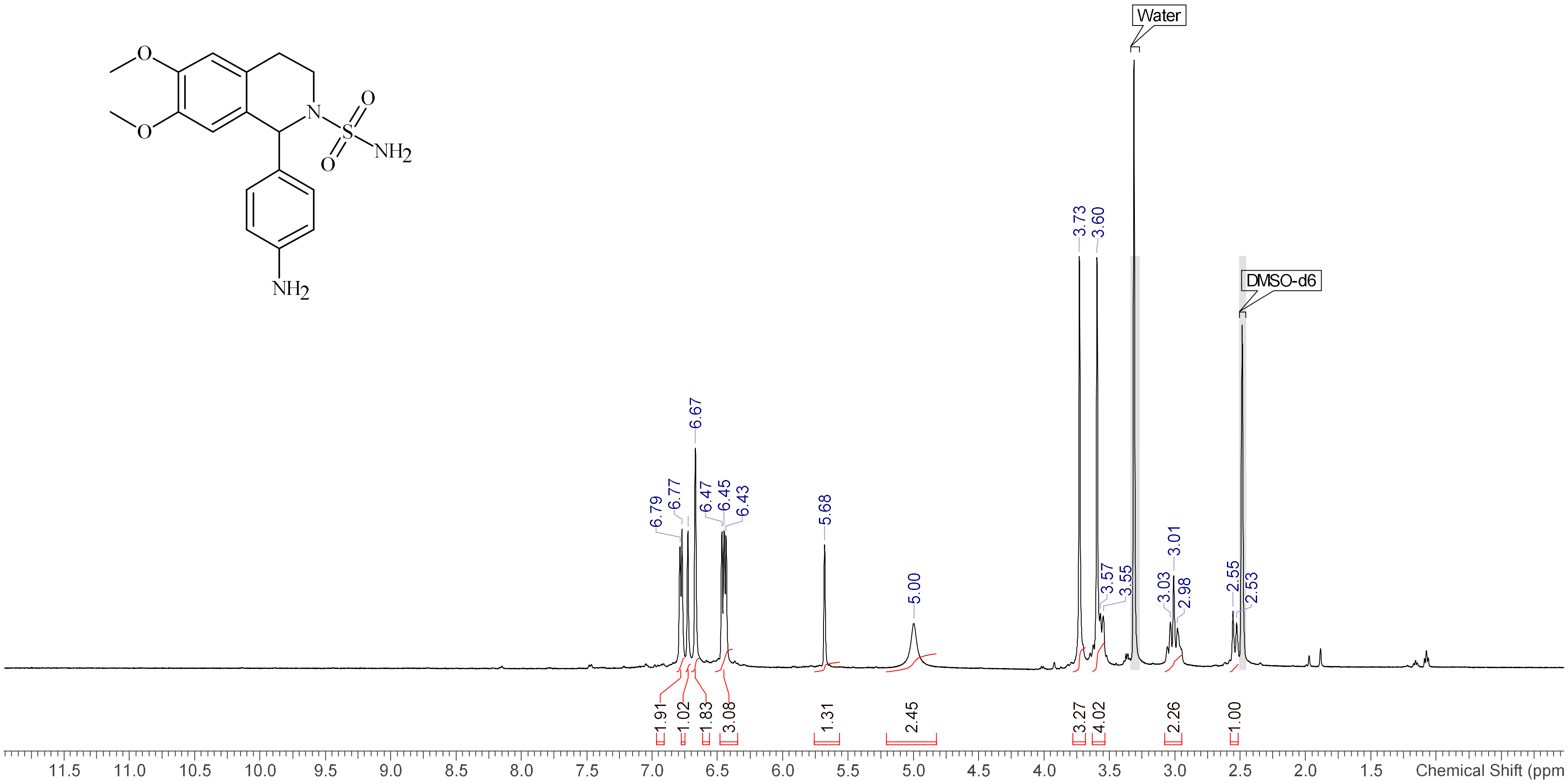

Figure S3: ^{1}H-NMR (DMSO-d_6) spectrum for 1-(4-aminophenyl)-6,7-dimethoxy-3,4-dihydroisoquinoline-2(1*H*)-sulfonamide (**3**)

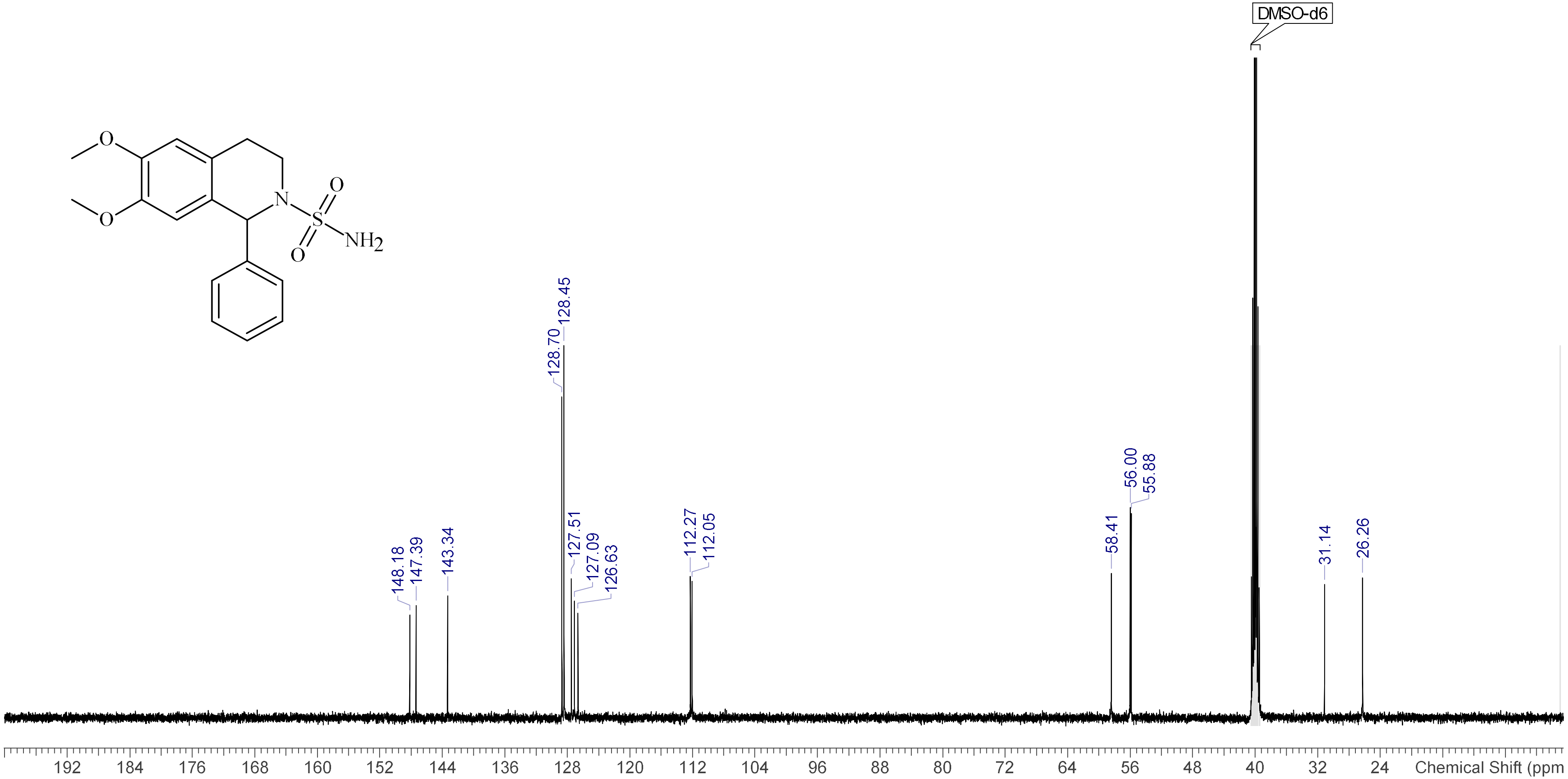

Figure S4: ^{13}C-NMR (DMSO-d_6) spectrum for 6,7-dimethoxy-1-phenyl-3,4-dihydroisoquinoline-2(1*H*)-sulfonamide (**1**)

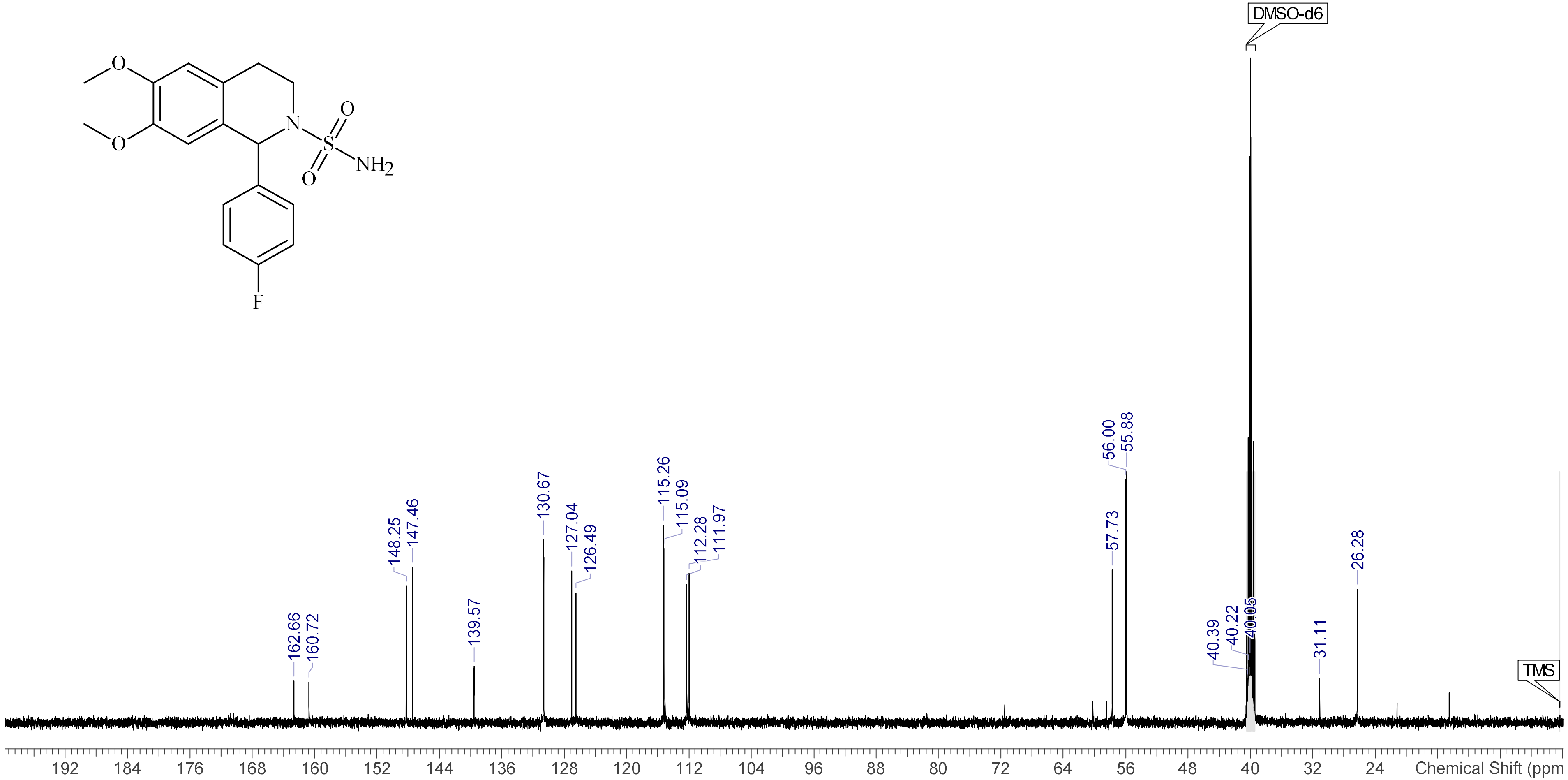

Figure S5: ^{13}C-NMR (DMSO-d$_6$) spectrum for 1-(4-fluorophenyl)-6,7-dimethoxy-3,4-dihydroisoquinoline-2(1*H*)-sulfonamide (**2**)

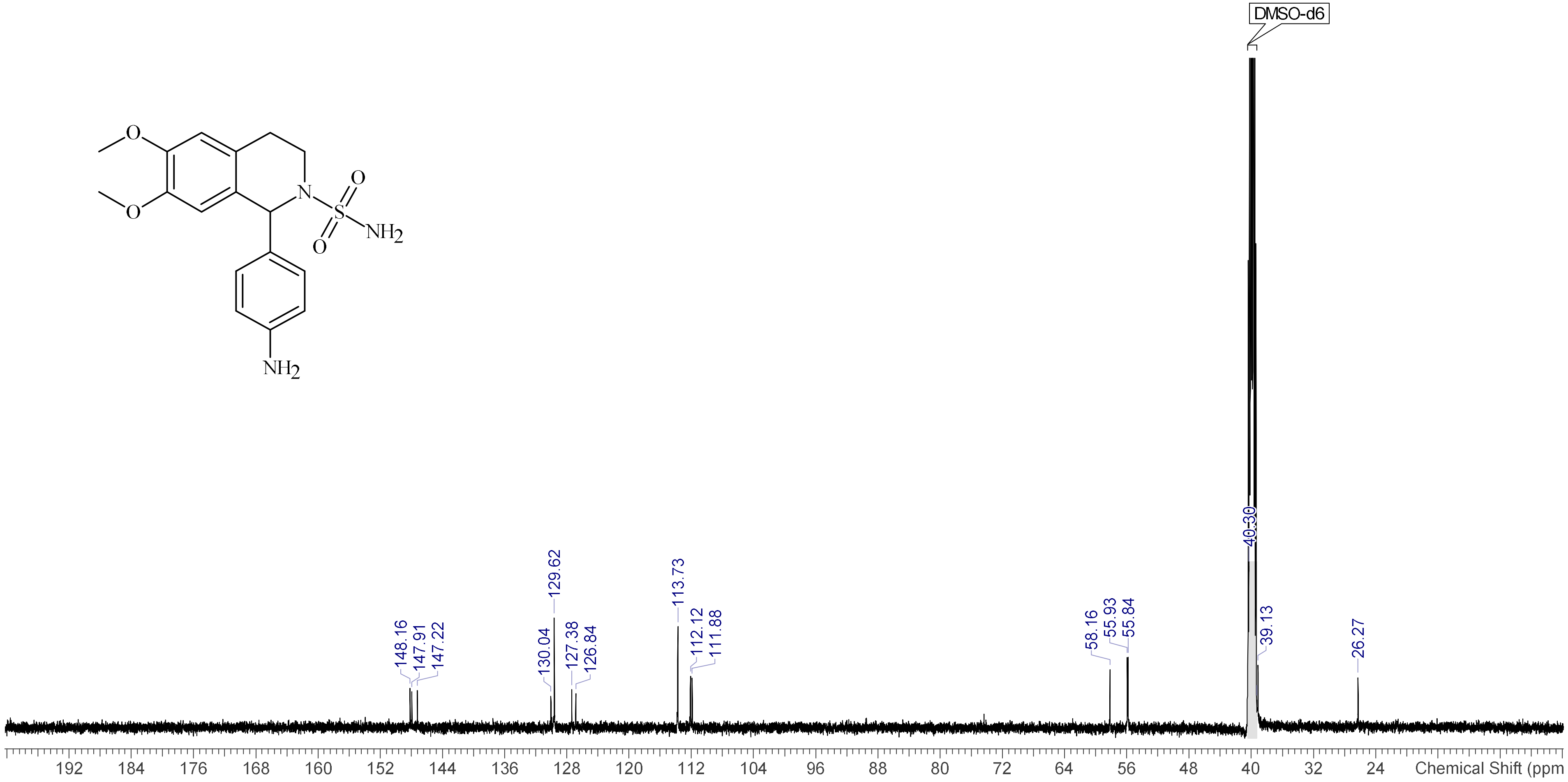

Figure S6: ^{13}C-NMR (DMSO-d_6) spectrum for 1-(4-aminophenyl)-6,7-dimethoxy-3,4-dihydroisoquinoline-2(1*H*)-sulfonamide (**3**)

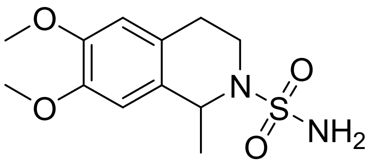

Figure S7. Chemical structure of 6,7-dimethoxy-1-methyl-3,4-dihydroisoquinoline-2(1*H*)-sulfonamide (**4**)

	K_i (μM)			
	**hCA I**	**hCA VII**	**hCA IX**	**hCA XIV**
**1**	8.98	7.1 x 10^{-3}	8.44	3.86
**2**	4.43	6.0 x 10^{-3}	5.59	6.93
**3**	8.07	6.2 x 10^{-3}	6.78	3.07

Table S1. *K*i values of compounds **1-3** against hCA I, hCA VII, hCA IX and hCA XIV. These data were previously reported in literature by us [1-3] and were included here for comparative purpose

References

[1] R. Gitto, S. Ferro, S. Agnello et al., "Synthesis and evaluation of pharmacological profile of 1-aryl-6,7-dimethoxy-3,4-dihydroisoquinoline-2(1H)-sulfonamides", *Bioorg Med Chem*, 17, (2009): 3659. https://10.1016/j.bmc.2009.03.066

[2] R. Gitto, F. M. Damiano, L. De Luca et al., "Synthesis and biological profile of new 1,2,3,4-tetrahydroisoquinolines as selective carbonic anhydrase inhibitors", *Bioorg Med Chem*, 19, (2011): 7003. https://10.1016/j.bmc.2011.10.015

[3] E. Bruno, M. R. Buemi, L. De Luca et al., "In Vivo Evaluation of Selective Carbonic Anhydrase Inhibitors as Potential Anticonvulsant Agents", *ChemMedChem*, 11, (2016): 1812. https://10.1002/cmdc.201500596

Table S2. Validation report of the ensemble docking protocol for hCA XII co-crystallized ligands. Each PDB structure was coloured according to the same scheme of Figure 5. The "Ligand" column reports the molecule ID of the respective co-crystallized ligand. RMSD values were calculated using two different methods, one considering the displayed heavy – atoms (v. atoms), the other one considering the maximum common structure (MCS). "RMS success" indicates docking poses with an RMSD value ≤ 2.6 Å relative to the X-ray conformation. The "Best receptor" column reports the ensemble receptor associated with the docking pose selected, corresponding to the representative structure of each cluster. "Ensemble success" indicates whether the ligand was docked into the representative structure of the cluster including its native protein. Tables were generated using Microsoft Office Excel (2016).

PDB	Ligand	RMSD (v. atoms)	RMSD (MCS)	RMS Success	Best Receptor	Ensemble success
1JD0	AZM	1.7663	1.4845	Y	3	N
4HT2	V50	3.2523	2.3647	Y	3	N
4KP5	E1F	1.3429	1.1299	Y	3	N
4KP8	E1G	2.6207	2.3699	Y	3	N
4Q0L	V14	2.1967	2.0946	Y	1	Y
4QJ0	WWX	6.2328	6.2181	N	3	N
4QJO	V1F	2.3241	2.1240	Y	1	Y
4QJW	WWO	1.3826	1.3826	Y	1	Y
4WW8	VD9	1.7984	1.5341	Y	2	Y
5LL5	6YH	2.2718	2.2718	Y	2	N
5LL9	6YQ	2.7461	2.7461	N	3	N
5LLO	5EF	1.6284	1.4957	Y	1	Y
5LLP	6Z9	5.6776	5.6631	N	3	N
5MSA	3TV	1.4824	1.2310	Y	2	Y
5MSB	V13	1.0366	0.6775	Y	2	Y
6G5L	EM5	1.4295	1.4295	Y	3	Y
6G7A	EOQ	1.8460	1.8460	Y	2	Y
6QN0	J8Q	2.4455	2.3392	Y	3	Y
6QNG	J95	1.8615	1.7572	Y	2	N
6QNL	J92	4.0770	3.9768	N	3	N
6R6Y	EA3	1.6793	1.1828	Y	3	N
6R71	JTW	1.5965	1.5965	Y	3	N
6T5P	VD8	1.8624	1.0794	Y	2	N
6T5Q	MKQ	0.6322	0.6322	Y	1	N
7PP9	7VZ	1.8672	1.7006	Y	3	Y
7PUU	840	1.4766	1.1614	Y	2	N
7PUV	84Z	1.6793	1.5647	Y	2	N
7PUW	84I	1.3682	1.3682	Y	1	N

Cluster 1 =
Cluster 2 =
Cluster 3 =

RMSD (v. atoms) = RMSD considering the displayed heavy-atoms (value in Å)

RMSD (MCS) = RMSD considering the maximum common structure (value in Å)

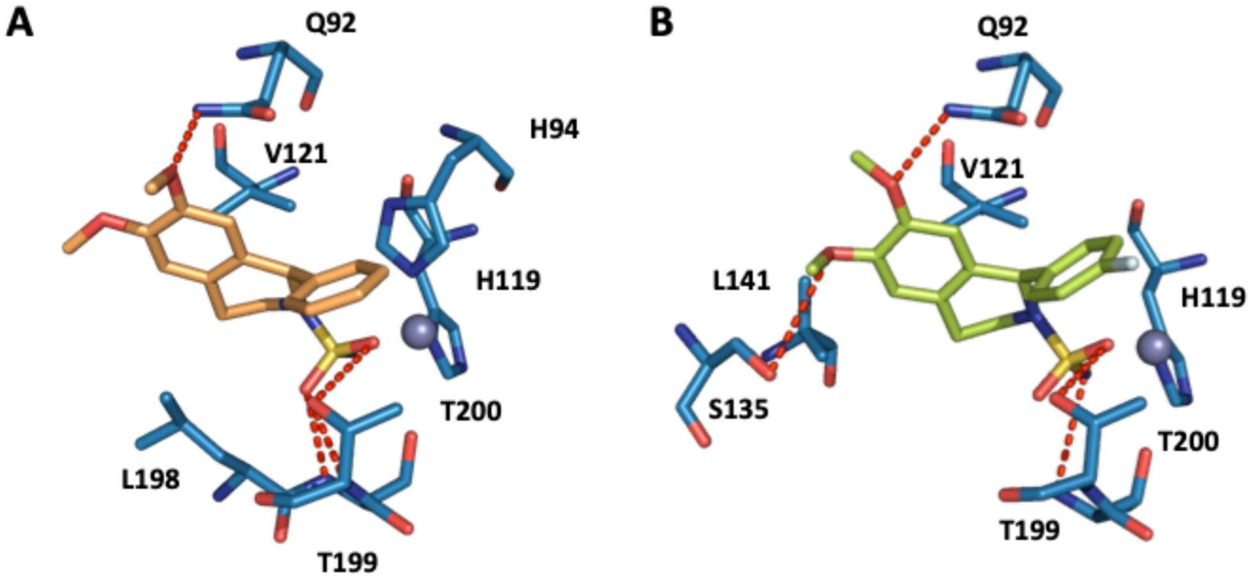

Figure S8. Predicted binding mode of (*R*)–**1** (A, orange sticks) and (*R*)–**2** (B, lemon sticks) to hCA XII. Hydrogen bond interactions are depicted as dashed red lines.

Docking analyses of derivatives *(R)*-**1** and *(R)*-**2** revealed that the anchoring of the sulfamide group to the catalytic zinc ion was conserved. The C–1 substituent of both molecules was positioned in the same region of hCA XII cavity as found for enantiomer (*S*)-**2**, forming hydrophobic interactions with T200, while the dimethoxybenzene-fused rings were oriented toward the hydrophilic region of the active site. An additional hydrogen bond interaction was observed between one methoxy group and Q92 side chain for both enantiomers.
